# Distal Tibial Metaphyseal Fractures: Does Blocking Screw Extend the Indication of Intramedullary Nailing?

**DOI:** 10.1155/2014/542623

**Published:** 2014-02-17

**Authors:** Mugundhan Moongilpatti Sengodan, Singaravadivelu Vaidyanathan, Sankaralingam Karunanandaganapathy, Sukumaran Subbiah Subramanian, Samuel Gnanam Rajamani

**Affiliations:** ^1^Coimbatore Medical College, Coimbaore 641018, India; ^2^Madras Medical College, Chennai 600001, India; ^3^Kilpauk Medical College, Chennai 600010, India

## Abstract

*Aim*. To evaluate the clinical use of blocking screws as a supplement to stability in distal tibial metaphyseal fractures treated with statically locked intramedullary nail. *Main Outcome Measurement*. Alignment and reduction preoperatively, postoperatively, and at healing were the main outcome measured with an emphasis on maintenance of initial reduction on followup. *Patients and Methods*. This was a prospective study of 20 consecutive cases of distal tibial metaphyseal fractures treated with statically locked intramedullary nailing with supplementary blocking screw between August 2006 and September 2007 with a maximum followup of 3 years. Medullary canal diameter was measured at the levels of fracture and isthmus. *Results*. The mean diameter of tibia at the level of isthmus was 11.9 mm and at the fracture site was 22.9 mm. Mean length of distal fracture segment was 4.6 cm. Mean varus/valgus alignment was 10.3 degrees preoperatively and 1.7 degrees immediatly postoperatively and was maintained till union. Using Karlstrom-Olerud score the outcome was excellent to good in 90%. *Conclusion*. We conclude that the use of blocking screw as a supplement will aid in achieving and maintaining the reduction of distal tibial metaphyseal fractures when treated with intramedullary nailing thereby extending the indication of intramedullary nailing.

## 1. Introduction

Treatment of metaphyseal fractures of tibia remains a challenge. The goals of surgical management include correction and maintenance of sagittal and coronal alignment, establishment of length and rotation, and early functional range of movements of knee and ankle.

Interlocking nailing of tibial fractures is desirable because this technique allows some load sharing, spares extra osseous blood supply, avoids extensive soft tissue dissection, and is familiar to most surgeons.

Nailing of metaphyseal fractures with short distal fragment is associated with an increase in malalignment particularly in coronal plane, nonunion, and need for secondary procedures to achieve union. The cause has been attributed both to displacing muscular forces and residual instability [1].

As there is a mismatch between the diameters of the nail and the medullary canal, with no nail-cortex contact, the nail may translate laterally along coronally placed locking screws and increased stress is placed on the locking holes to maintain fracture alignment after surgery [1].

Various techniques have been recommended to improve nailing the metaphyseal fractures including blocking screws (poller screw), temporary unicortical plating, percutaneous reduction clamps, and fibular plating.

## 2. Blocking Screw 

Blocking screws placed adjacent to the nail and perpendicular to the screw holes usually in an anteroposterior direction had been suggested as one possible method of improving the stability of metaphyseal fractures and had been described as a reduction tool used to overcome the displacing forces at the time of introduction of intramedullary nail.

The blocking screws functionally decrease the width of the metaphyseal medulla and are particularly useful with nails of smaller diameter [1].

In 1994 Krettek et al. described the clinical application of blocking screws, termed poller screws, as a tool for the prevention of axial deformities of proximal and distal third fractures of tibia during intramedullary nailing [2, 3].

## 3. Patients and Methods

This was a prospective study of 20 cases of distal tibial metaphyseal fractures treated with statically locked intramedullary nailing with supplementary blocking screws between August 2006 and September 2007 at our institute with a minimum followup of 5 years.

Displaced distal tibial metaphyseal fractures in adults treated with intramedullary nailing were included in the study. The fractures included were acute fractures and delayed union. Both open and closed fractures were included in the study.

Tibial diaphyseal and proximal metaphyseal fractures were excluded from the study. Metaphyseal fractures treated with statically locked intramedullary nails but with additional procedures like fibular plating were also excluded from the study.

Among the operatively treated 65 tibial fractures about 20 cases met the inclusion criteria.

There were 16 males and 4 female patients with a mean age of 37.75 years (95% lower confidence limit of (LCL) 33.13 years and 95% upper confidence limit of (UCL) 42.36). The injury was on the right side in 12 cases.

The mechanism of injury was road traffic accident in all except three in whom it was fall from height in two and fall of a heavy object over the leg in one.

According to AO guidelines there were five type 43 A1, eleven type 43 A2, and four type 43 A3 fractures.

Injury was closed in 15 fractures and Gustilo Anderson grade I in 2 and grade II in 3 patients.

The mean distance from the articular surface was 4.6 cm (95% LCL 2.8 cm and 95% UCL 5.5 cm) and the mean length of the fracture was 3.4 cm (95% LCL 2.69 cm and 95% UCL 4.10 cm).

The mean delay between the injury and the surgery was 3.75 weeks (95% LCL 1.23 weeks and 95% UCL 6.26 weeks). Among the 20 cases two were delayed union of 18-week duration.

The mean operating time was 75 minutes. The mean diameter of the medullary canal at the level of isthmus was 11.9 mm and at the fracture site was 22.9 mm (Table 1).

The mean length of distal metaphysis was 5 cm.

### 3.1. Blocking Screws 

Blocking screws were selected for use for one or more of the following reasons:

1. to correct alignment after insertion of nail (8 fractures),
2. to maintain alignment or to improve the stability of bone implant complex (20 fractures),
3. to control the nail during insertion (5 fractures).

In 7 cases single blocking screw was used on the concave side of the deformity, close to the fracture site in the short fragment. In 2 cases single blocking screw was used on the convex side of the deformity, near the end of the nail. And in the rest of cases 2 blocking screws were placed, the first one on the concave side of the deformity close to the fracture site and the second screw on the convex side of deformity near the end of the nail in the distal fragment.

Depending on the amount of correction needed, the screws used for blocking were 4 mm locking screws or 4.5 mm cortical screws.

### 3.2. Preoperative Planning 

X-ray of the injured leg in AP and lateral views was taken. The fracture tendency for valgus or varus and antecurvatum or recurvatum malalignment was noted. The angle of malalignment was measured.

Fracture was classified according to AO. Fracture location from distal articular surface and the length of fracture were measured. The diameters of medullary canal at isthmus and at the level of fracture were also measured.

Appropriate length of the nail was measured in the contralateral leg, from the tibial tuberosity to medial malleolus. Open fractures were dealt with according to AO principles.

### 3.3. Operative Technique 

Metaphyseal fractures were stabilized with statically locked intramedullary nails on a standard radio lucent table with manual traction. All cases were done under spinal anesthesia. Tourniquet was not used in any case. Through patellar tendon splitting approach, entry point was made in the midline. Guide wire was passed under image intensifier control.

Closed reduction was done in all except two fractures. In those fractures, closed reduction was attempted and we had to do open reduction as there was a marked overriding of the fragments due to a delay of 18 weeks before surgery.

The nails used were unreamed cannulated stainless nails, with 2 proximal (mediolateral) and 3 distal (2 mediolateral and 1 anteroposterior) locking options, of diameter 8 or 9 mm. In one case the tibia was too narrow and too short where we have used a nail of 7 mm diameter.

The blocking screw was used on the concave side of the deformity close to the fracture in the short fragment when single screw was used between the cortex and the nail under image intensification. When 2 blocking screws were placed, the second screw was on the convex side of deformity near the end of the nail in the short fragment. But in 2 cases the distal segments were too short and there was significant comminution on the concave side of the deformity. Hence we used single poller screw on the convex side of deformity near the tip of the nail.

In cases of malalignment and instability the screw holes were drilled with the nail in place while applying manual overcorrection. 2.5 mm or 3 mm K wire was used to drill the pilot hole for poller screw as the drill bit may damage the nail while drilling with the nail in situ.

**Figure 1:**
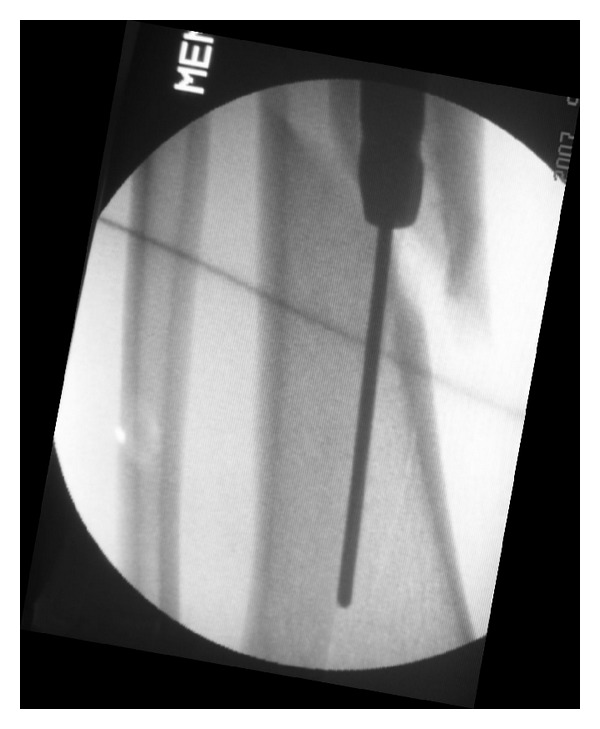
Intraoperative picture showing varus alignment with nail in situ.

**Figure 2:**
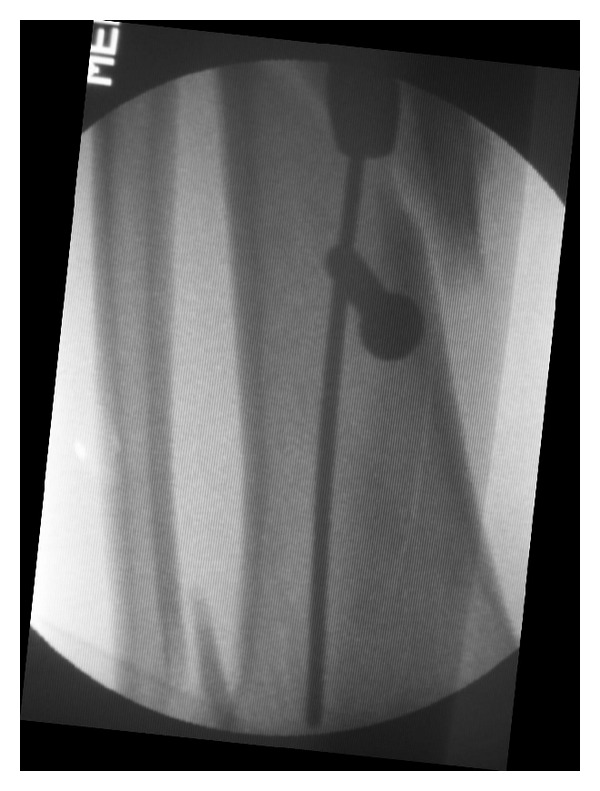
Poller screw placed on the concave side close to the fracture in the distal segment.

For fractures which were stable but malaligned, the nail was temporarily removed, the blocking screws were placed, and the nail was reinserted (Figures 1 and 2).

**Figure 3:**
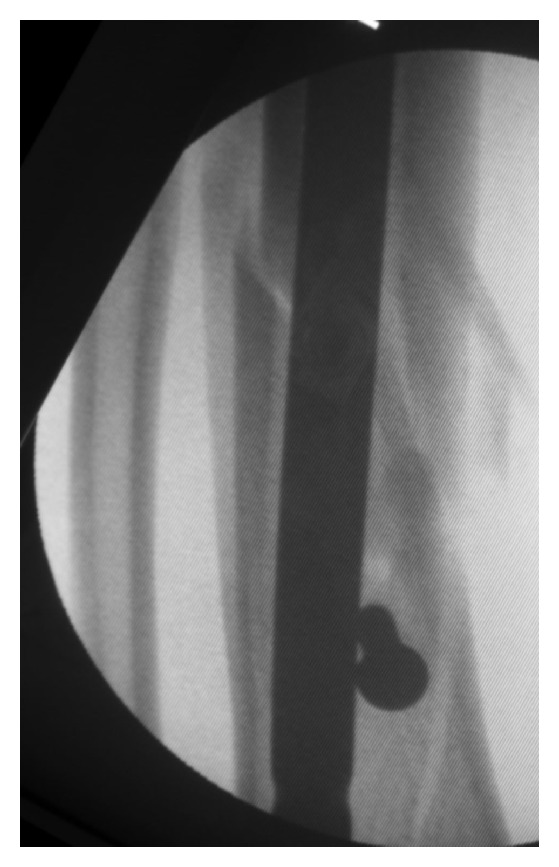
Alignment restored as the nail is introduced.

Distal and proximal locking was done after achieving the alignment using blocking screws (Figure 3). The alignment was confirmed in both coronal and sagittal plane with image intensifier.

### 3.4. Postoperative Treatment 

Partial weight bearing was started in the second postoperative week in all except two cases. In one where we have used 7 size nail, we recommended non-weight-bearing till radiological evidence of union and in the other where tibialis anterior tendon was found cut and the patient had both bones fractures in the contralateral leg, partial weight bearing could not be started. In both the cases cast support was given for 4 weeks.

Partial weight bearing continued up from 4 to 8 weeks; thereafter full weight bearing started depending on clinical and radiological evidence of union.

### 3.5. Followup 

All the fractures were followed through till union of fracture with clinical and radiological examination at intervals of 4 to 6 weeks. The maximum followup was 3 years.

On followup axial alignment was assessed and functional analysis was quantified using Karlstorm-Olerud score. Valgus and antecurvatum were expressed as positive values and varus and recurvatum were expressed as negative values.

Radiographs were analyzed for correction, maintenance of position, or loss of reduction. Shortening and rotational malalignment were not measured.

Fracture was defined as united when patient was able to bear full weight on the injured limb without pain and without support and when radiographs showing bridging call us in at least 3 cortices.

### 3.6. Data Analysis 

Repeated measures ANOVA test was used to analyze the results [4]. Karlstorm-Olerud score was used to assess the functional outcome. It is an independent measurement, not influenced by other comorbid conditions and associated injuries [5].

## 4. Results

All the relevant data were analyzed. All the fractures eventually united in a mean period of 11.5 weeks (95% LCL 10.11 weeks and 95% UCL 12.88 weeks). Karlstrom-Olerud score was excellent in 14 fractures (70%), good in 4 patients (20%), and fair in 2 patients (10%).

Radiologically the mean postoperative varus/valgus alignment was ±1.7 degrees (95% LCL 0.5 degrees and 95% UCL 2.9 degrees) when compared to the mean preoperative varus/valgus alignment of ±10.3 degrees (95% LCL 8.2 degrees and 95% UCL 12.4 degrees).

The alignment was maintained till union with the mean remaining the same in the coronal plane. Repeated measures ANOVA test showed the *F*-test value of 45.29 which is significant as the *P* value is 0.00000 (*P* < 0.05).

The mean postoperative antecurvatum/recurvatum alignment was ±0.2 degrees (95% LCL −0.1 degrees and 95% UCL 0.5 degrees) when compared to the mean preoperative antecurvatum/recurvatum alignment of ±8.0 degrees (95% LCL 4.6 degrees and 95% UCL 11.3 degrees). *F*-test value in repeated measures ANOVA is 22.845 with a *P* value of 0.0000 (<0.05) which is statistically significant. The mean antecurvatum/recurvatum alignment was maintained till union at ±0.2 degrees. The mean ratio of fracture segment to the nail length (i.e., the length of tibia) was 14%.

**Figure 4:**
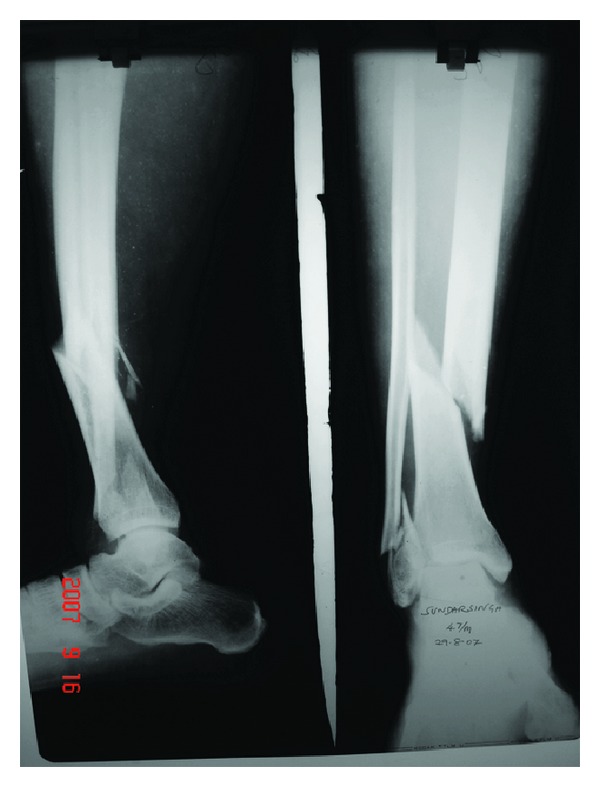
Preoperative X-ray showing varus and antecurvatum.

**Figure 5:**
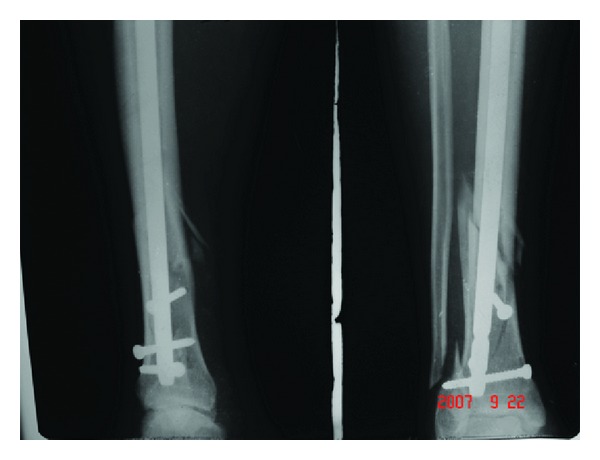
Immediate postoperative X-ray showing good alignment in spite of additional fracture line due to poller screw.

**Figure 6:**
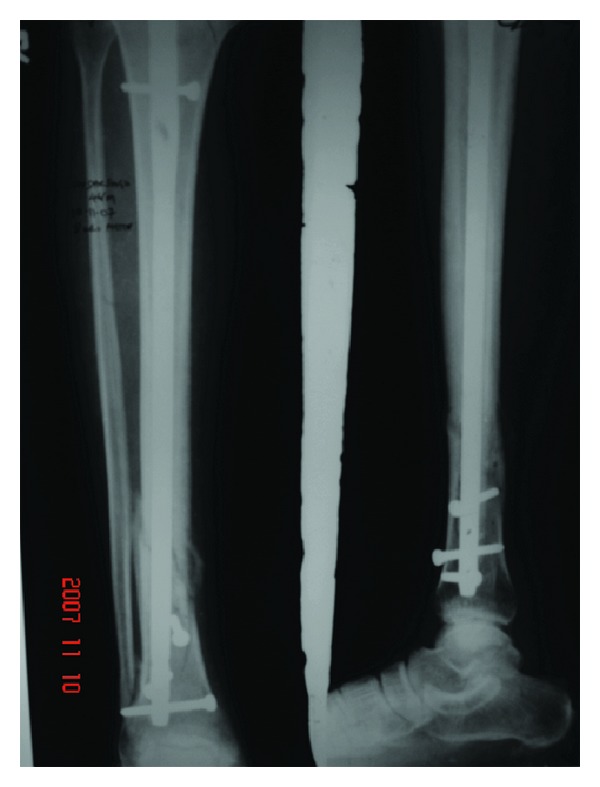
Eight-week postoperative X-ray showing fracture union without any loss of reduction.

The poller screw related complication was encountered in one case where we had new fracture lines while introducing the nail after placement of poller screw (Figures 4 and 5). But the alignment was achieved and maintained and the fracture united within 8 weeks (Figure 6).

Complications which were not related to poller screw were encountered in two cases. Both of them had developed deep infection and went in for delayed union of which one required dynamisation to achieve union. No complications of nerve injury or compartment syndrome were encountered. There were no incidences of breakage of nail, locking screw, or blocking screw.

## 5. Discussion

We cannot overemphasize the potential advantages of intramedullary nailing more than any other form of fixation like external fixator or plating in tibial fractures. But the problems in extending the indications to metaphyseal fractures have to be analyzed and resolved.

The amount of malalignment and shortening considered acceptable is controversial [6]. Tarr et al. and Puno et al. demonstrated that distal tibial malalignment may be more poorly tolerated than proximal malalignment [7].

Trafton's recommendation is generally agreed on by many authors. As per Trafton's recommendation the acceptable malalignment is less than 5 degrees of varus-valgus angulation, 10 degrees of anteroposterior angulation, 10 degrees of rotation, and 15 mm of shortening [7]. In our study we encountered malalignment in two cases (10%).

Merchant and Dietz in 1989 suggested that for tibial fractures deformity of >5° was associated with radiographic changes in the ankle [6].

Van der Schoot reported a 15-year followup of 88 patients with fractures of lower leg. 49% had healed with malalignment of at least 5 degrees. More arthritis was found in the knee and ankle adjacent to fracture than in comparable joints of the uninjured leg. Malaligned fractures showed significantly more degenerative changes [8]. Puno et al. recorded the long-term effects of tibial angular malunion on knee and ankle joints in his 28 tibial fractures with an average followup of 8.2 years. His analysis showed greater degrees of ankle malalignment producing poorer clinical results [9].

Kyro A in his series of 64 tibial shaft fractures concluded that malunion of tibial shaft fractures seems to be especially harmful in distal fractures, in fractures with marked previous displacement, in fractures caused by high-energy injury, and among patients less than 45 years of age [10]. Ahlers and Von Issendorf analyzed 386 fractures of tibia treated by intramedullary nailing of which 32 were proximal and 138 were distal third fractures. In both the groups one-quarter to one-third had varus-valgus deformities greater than 4 degrees [11]. In another study, Mosheiff in 1999 found that 42% of distal third fractures treated with interlocking nailing required secondary procedures to achieve union [12].

There has been discrepancy in the literature regarding the locking bolt orientation and its effect on fracture nail construct stability.

Chen compared the intrinsic stability in tibial intramedullary nail constructs in distal third diaphyseal fractures without isthmal support, between two mediolateral distal locking screws and two perpendicular (one mediolateral and one anteroposterior) distal locking screws. He concluded that fixation stability of intramedullary nail is not significantly influenced by distal locking screw orientation in response to sagittal, coronal, or rotational forces [13]. On the contrary, Smucker et al. found two parallel locking bolts being a better construct than perpendicular locking bolts in their study [14].

We have analyzed the mismatch between the diameters of medullary canal at the level of isthmus (i.e., maximum possible nail size) and at the fracture site in all cases. We found that there was a significant *P* = 0.0000 (*P* < 0.5) mismatch between them. The mean diameter of medullary canal at the level of isthmus was 11.9 mm compared to 22.9 mm at the level of fracture site. This mismatch explained the cause of instability in metaphyseal fractures when treated with intramedullary nailing. To overcome the issue of malalignment various techniques have been developed.

In distal third fractures, fibular plating, one cross screw across fracture site as lag screw, use of large reduction forceps, temporary unicortical plating, percutaneous manipulation with Shanz pins, femoral distracter and cutting the distal few millimeters of nail distal to the distal screw hole to allow two cross locking screws in the distal fragment, have been the supplementary procedures used to achieve the alignment [14–24].

The primary aim of the study was to analyze the effectiveness of achieving and maintaining reduction in metaphyseal fractures of tibia treated with intramedullary nailing using supplementary blocking screws.

We have also measured the maximum diameter of the metaphysis distal tibia, thereby the length of the distal metaphyseal segment in our population was reached. The mean length of distal metaphysis was 5.0 cm.

As described in various literatures the malalignment in these circumstances was significantly high when done without any supplementary procedures.

Krettek et al. in 1999 published the mechanical effect of blocking screw in stabilizing tibial fractures with short proximal or distal fragments after insertion of small diameter intramedullary nails. Krettek et al. created bone implant constructs (BIC) in fresh cadaveric tibiae and demonstrated in distal BICs the addition of blocking screws decreasing the average deformation of the BICs by 57% (*P* < 0.0001) [25, 26].

Ai et al. explored the effect of blocking screws on the breakage of interlocking intramedullary nails and concluded that blocking screws improve the stability of fracture area distinctively and hence reduce the breakage of intramedullary nailing [27]. According to James Kellam, meticulous intramedullary techniques combined with the use of fibular plate fixation or blocking screws will achieve the best results in maintaining the reduction of distal tibial fractures till union [17].

The use of blocking screw as reduction tool was established in our study by the repeated measures ANOVA test and was comparable to the study by Krettek.

Blocking screws improved the stability of the metaphyseal fractures after nailing and promoted union in our study. Secondary procedure was required in only one case to achieve union (5%). Dynamisation was done 6 weeks after interlocking nailing that developed deep infection. The fracture was originally a grade II compound fracture treated with external fixator which was removed once the wound healed. Nailing was done 6 weeks after removal of fixator.

No cases required bone grafting, bone marrow injection, or exchange of nailing.

The ratio of short metaphyseal fragment length to the total tibial length was analyzed. The total length of the tibia was approximately derived from the length of the nail used. The mean ratio was found to be 14%.

This indicates that even such short metaphyseal fragments had been effectively stabilized till union with intramedullary nailing when supplemented with blocking screw.

Blocking screws functionally reduce the width of the metaphyseal medulla, and usually blocking screw is applied in anteroposterior direction as the coronal plane malalignment is more prone to occur than the sagittal plane. Moreover deformities in the sagittal plane are better tolerated and are less common if the fracture is reduced at the time of initial locking. But when the fracture pattern suggests instability in sagittal plane, blocking screw should be used in the mediolateral direction.

Paige Whittle A and George W Wood II in their analyses of the influence of fibular fractures on maintaining alignment in 40 distal tibial fractures treated with locked intramedullary nailing concluded that 60% of unfixed fibular fractures occurring at the same level as the tibial fracture were malaligned.

In our study, fibular fracture was associated in all but one patient. It was at the same level of tibial fracture in 18 cases of which 2 were segmental and distal to tibial fracture in two cases. Only 11% (2/18) of unfixed fibular fractures occurring at the same level as the tibial fractures were malaligned, which is statistically not significant. We found that in interlocking nailing of distal third tibial fractures, when supplemented with blocking screw, level of fibula fracture did not influence the stability or the functional outcome.

When compared to other techniques described for preventing metaphyseal malalignment during nailing, blocking screws are technically easy and reproducible, do not require any special instrumentation, and do not need any special design modifications in the nail. There is no need for excessive soft tissue dissection or additional hardware like unicortical plating or fibular plating. There is no significant increase in radiation exposure for applying blocking screws.

We had excellent to satisfactory outcome in 90% by Karlstrom-Olerud scoring which is comparable to the results of Krettek et al. with 94% excellent to satisfactory outcome.

Limitations in our study include small number of cases and lack of control group.

## 6. Conclusion

We conclude that blocking screw by acting as a reduction tool, functionally reducing the medullary width and preventing the loss of initial reduction, definitely extends the indication of intramedullary nailing to distal tibial metaphyseal fractures.

## Figures and Tables

**Table 1:** Medullary canal diameter (in mm).

	Mean	S.D	95% LCL	95% UCL
Isthmus	11.9	1.7	11.1	12.7
Fracture site	22.9	6.6	19.8	25.9
Distal metaphysis	50.2	3.5	48.5	51.8
